# Dementia with Lewy bodies and additional progressive supranuclear palsy presenting with early postural instability and frequent falls: an autopsy case

**DOI:** 10.1186/s12883-026-04704-z

**Published:** 2026-02-17

**Authors:** Hideyuki Moriyoshi, Naoki Hayashi, Akio Akagi, Yuichi Riku, Jun Sone, Akiko Yoneyama, Hiroaki Miyahara, Masahisa Katsuno, Hisayoshi Niwa, Yasushi Iwasaki

**Affiliations:** 1 Department of Neuropathology, Institute for Medical Science of Aging, Aichi Medical University, 1-1 Yazakokarimata, Nagakute, Aichi 480-1195 Japan; 2 Department of Neurology, Nagoya University Graduate School of Medicine, Nagoya, Japan; 3 Department of Neurology, Kariya Toyota General Hospital, Kariya, Japan; 4 Department of Pathology, Kariya Toyota General Hospital, Kariya, Japan

**Keywords:** Dementia with Lewy bodies, Progressive supranuclear palsy, Postural instability, Mesencephalic locomotor region

## Abstract

**Background:**

Dementia with Lewy bodies (DLB) is characterized by fluctuating cognition, visual hallucinations, and Parkinsonism, whereas progressive supranuclear palsy (PSP) typically presents with supranuclear gaze palsy, postural instability, and akinesia or rigidity. Although they represent distinct pathological entities, comorbid Lewy body disease and PSP has occasionally been reported and may contribute to clinical heterogeneity. We report an autopsy case of comorbid DLB with mild PSP pathology, clinically manifesting as DLB with early onset falls, resulting in cerebral contusion and traumatic subarachnoid hemorrhage.

**Case presentation:**

An 86-year-old patient developed progressive memory impairment 1 year before death, followed by cognitive fluctuations, visual hallucinations, and delusional speech. Two months before death, the patient required wheelchair assistance due to gait disturbances and frequent falls. Neurological examination revealed tremors and rigidity in both upper limbs. Computed tomography of the head revealed cerebral contusion, traumatic subarachnoid hemorrhage, and chronic subdural hematoma. The clinical presentation was consistent with that of DLB, although the patient experienced prominent falls and marked postural instability. The patient died at the age of 87, soon after initial consultation. Neuropathological analysis revealed the presence of Lewy bodies in the substantia nigra and amygdala, with α-synuclein pathology in the brainstem and limbic system, consistent with limbic-predominant DLB. Tau pathology, including neurofibrillary tangles and tufted astrocytes, was present in the frontal lobe, globus pallidus, putamen and midbrain tegmentum, fulfilling the diagnostic criteria for PSP, although the overall severity was mild. α-synuclein pathology was also identified in the midbrain tegmentum including the cuneiform nucleus, a key component of the mesencephalic locomotor region.

**Conclusions:**

This case illustrates that comorbid DLB and PSP can produce an atypical phenotype showing core features of DLB, early severe postural instability, and frequent falls. We believe that dual involvement of tau and α-synuclein pathologies in the mesencephalic locomotor region in the midbrain tegmentum contributed to gait disturbances and falls, leading to cerebral contusion and traumatic subarachnoid hemorrhage.

## Background

Dementia with Lewy bodies (DLB) is characterized by progressive cognitive decline, fluctuating cognition, and visual hallucinations [1]. A key pathological feature of DLB is the presence of α-synuclein pathology in the limbic regions and cerebral cortices [1]. DLB shares an underlying pathology with Parkinson’s disease and presents with Parkinsonism, including rigidity, bradykinesia, resting tremor, and postural instability, as part of Lewy body disease (LBD) [1]. Progressive supranuclear palsy (PSP) typically presents with supranuclear gaze palsy, prominent postural instability, and symmetric akinesia or rigidity in the proximal regions, although various clinical subtypes have been defined [2]. PSP is characterized by 4-repeat tau pathology in neurons, astrocytes, and oligodendroglia of the subcortical nuclei [3]. Postural instability is a common clinical feature of both DLB and PSP; however, frequent early-onset falls are considered a hallmark of PSP and serve as an important distinguishing sign between PSP and LBD [4]. Although PSP and LBD represent distinct pathological diseases, their coexistence may contribute to the observed clinical heterogeneity [5, 6]. Herein, we present an autopsy case showing coexistence of DLB and mild PSP, which clinically manifested as DLB with prominent early-stage falls resulting in cerebral contusion and traumatic subarachnoid hemorrhage. This case exhibited a distinctive clinical presentation combining the core features of DLB, such as visual hallucinations and fluctuating cognition, with marked postural instability and a pronounced tendency to fall in the early stages of dementia.

## Case presentation

**Fig. 1:**
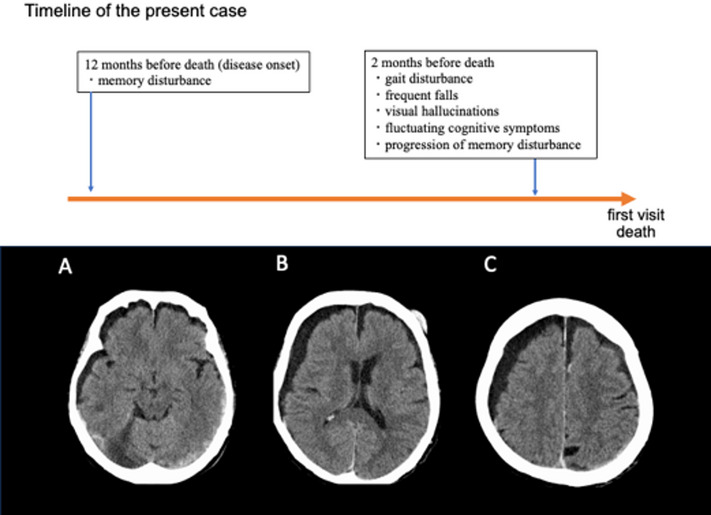
Timeline of the present case and brain computed tomography. The upper panel shows the timeline of the present case, and the lower panel shows brain CT images (**A**–**C**). **A** Midbrain atrophy was not notable. Traumatic subarachnoid hemorrhage was observed primarily in the left occipito-temporal lobe. **B** A subcutaneous hematoma was observed in the left frontal region. **B**, **C** A right chronic subdural hematoma was observed, with slight medial shift of the right cerebral hemisphere

An 86-year-old woman developed memory impairment approximately 1 year before death. The patient had been independent in activities of daily living before the onset of cognitive symptoms. Two months before death, the patient developed gait disturbances and frequent falls and required wheelchair assistance. Memory disturbances worsened and the patient developed visual hallucinations, confusion during conversation, and fluctuating cognitive symptoms. Four days prior to death, the patient visited the neurology department for the evaluation of dementia and gait disturbance. A frontal scalp hematoma and an occipital contusion, both resulting from frequent falls, were identified. Neurological examination revealed tremors and rigidity in the upper limbs. Because of postural instability, the patient easily fell both forward and backward. Computed tomography of the head revealed cerebral contusion, traumatic subarachnoid hemorrhage, and chronic subdural hematoma. Diffuse mild cerebral atrophy was observed, but no atrophy of the midbrain tegmentum was identified (Fig. 1A − C). A diagnosis of DLB was considered, and further investigation was planned. The cerebral contusions and traumatic subarachnoid hemorrhages were managed conservatively. However, the patient later collapsed at home, was transported to the emergency department, and subsequently died at the age of 87. Because of the unexpected clinical course, no further neurological assessments could be performed, and neither MRI nor nuclear imaging studies were obtained. An autopsy was performed with the consent of the family of the patient. Although the exact cause of death could not be determined, general pathological findings revealed pulmonary and hepatic congestion, suggesting acute heart failure as a possible cause.

### Neuropathological findings

**Fig. 2:**
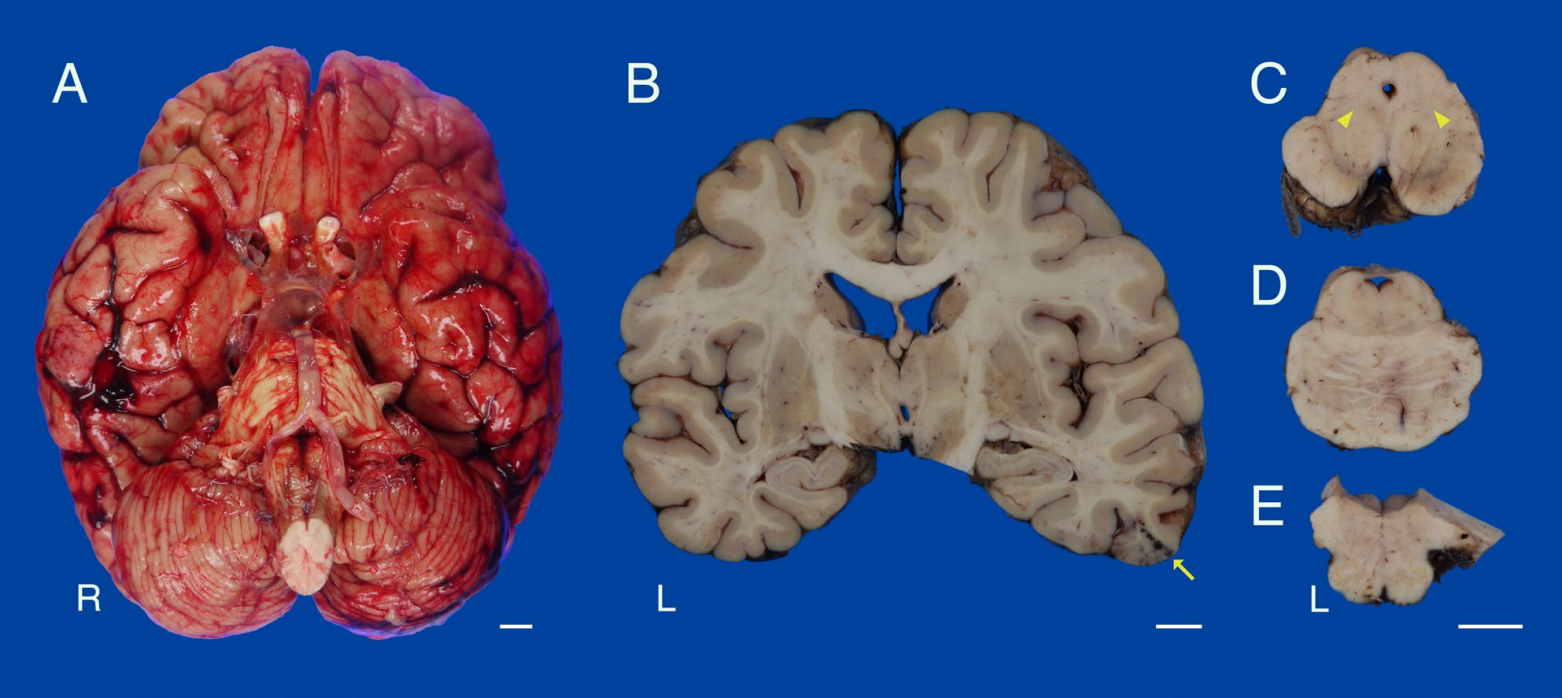
Brain macroscopic findings. **A** Basal surface of the brain. Subarachnoid hemorrhage was present in the bilateral temporal sulci. No brain herniation was detected. **B** Coronal section of the cerebrum. The globus pallidus, putamen, and subthalamic nucleus were preserved. Cerebral contusion was identified in the right inferior temporal gyrus (arrow). **C**–**E** Horizontal section of the brainstem. Depigmentation was noted in the substantia nigra and locus coeruleus (**C**, **E**). Brainstem atrophy was not observed. **C** The location of the cuneiform nucleus is indicated by arrowheads. Scale bar = 1 cm

**Fig. 3:**
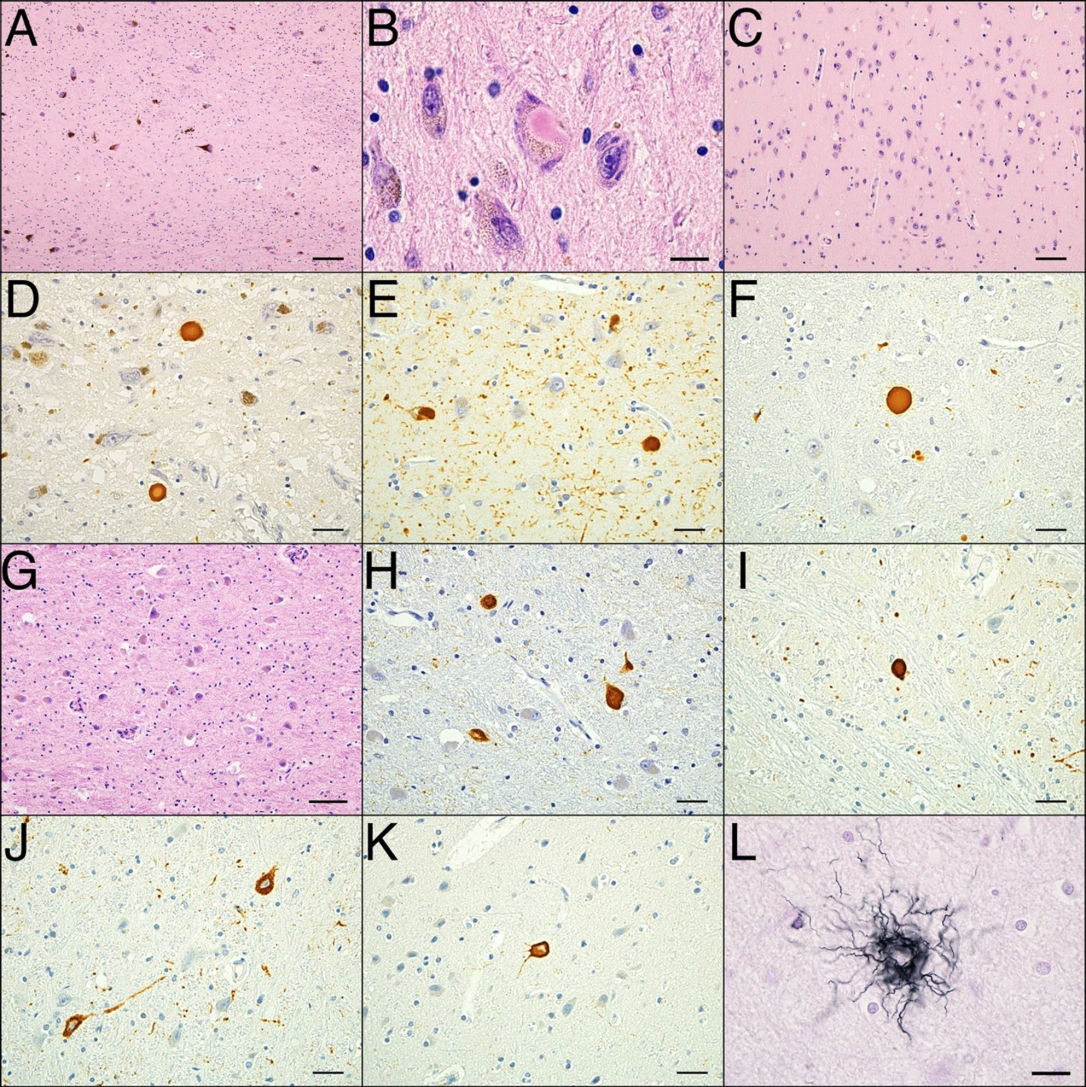
Brain microscopic findings. (A–C, G) Hematoxylin and eosin (H&E) staining. (D–F) Immunohistochemistry (IHC) against phosphorylated α-synuclein. (H–K) IHC against phosphorylated tau. (L) Gallyas–Braak staining. (**A**) Significant neuronal loss in the substantia nigra. (**B**) Brainstem-type Lewy bodies in the substantia nigra. (**C**) Spongiform changes in the transentorhinal region. (D–F) A notable number of α-synuclein-positive structures in the substantia nigra (**D**), transentorhinal region (**E**), and cuneiform nucleus of midbrain tegmentum (**F**). (**G**) H&E staining showed no apparent neuronal loss or astrogliosis in the subthalamic nucleus. (H–J) Moderate number of neurofibrillary tangles and coiled bodies in the subthalamic nucleus (**H**), globus pallidus (**I**), and cuneiform nucleus of the midbrain tegmentum (**J**), with only a few in the superior frontal gyrus (**K**). (**L**) Tufted astrocytes observed in the midbrain. Scale bar = 100 μm (A, C), 50 μm (G), 20 μm (D–F, H–K) and 10 µm (B, L)

The brain weighed 1220 g. Macroscopically, the brain showed mild diffuse cerebral atrophy. Subarachnoid hemorrhages were present in the right central sulcus, postcentral sulcus, and bilateral temporal sulci. There was no brain herniation (Fig. 2A). The globus pallidus, putamen, and subthalamic nucleus were grossly preserved (Fig. 2B). A cerebral contusion was identified in the right inferior temporal gyrus (Fig. 2B). Depigmentation was observed in the substantia nigra and locus coeruleus (Fig. 2C − D). No significant brainstem atrophy was observed (Fig. 2C − E). A chronic subdural hemorrhage was observed in the dura mater. Histologically, the dorsal motor nuclei of the vagus and the locus coeruleus exhibited severe neuronal loss and astrogliosis. The substantia nigra showed moderate neuronal loss and astrogliosis (Fig. 3A). Brainstem-type Lewy bodies were observed in the locus coeruleus and substantia nigra (Fig. 3B), and cortical-type Lewy bodies were found in the cingulate gyrus and amygdala. Moderate spongiform changes were observed in the transentorhinal region, amygdala, and cingulate gyrus (Fig. 3C). Immunohistochemistry (IHC) against phosphorylated α-synuclein (1:6000, mouse monoclonal, pSyn#64; Wako Pure Chemical Industries, Osaka, Japan) revealed a significant number of immunopositive structures in the brainstem, limbic regions, and spinal cord, including the cuneiform nucleus of the midbrain tegmentum (Fig. 3D–F). A small number of α-synuclein–positive inclusions were observed in the oculomotor nucleus and the Edinger–Westphal nucleus. Only a few α-synuclein-positive structures were observed in the neocortex. α-synuclein pathology was also observed in the sympathetic ganglia although neuronal loss was not notable. The distribution of α-synuclein pathology corresponded to the limbic-predominant type according to the DLB consortium classification (Table 1) [1].

**Table 1:** Summary of the distribution of Tau and α-synuclein pathology

		Tau	Tufted astrocyte	α-synuclein
Neocortex	Precentral gyrus	+	+	0
	Temporal cortex	0	0	+
	Parietal cortex	0	0	+
Limbic system	Entorhinal cortex	++	0	+++
	Amygdala	++	0	+++
Subcortical nuclei	Putamen	+	+	+
	Globus pallidus	+	0	+
	Subthalamic nucleus	++	0	+
Midbrain	Substantia nigra	++	0	+++
	Oculomotor nucleus	+	0	++
	Red nucleus	++	0	++
	Superior colliculus	++	+	++
	Tegmentum	++	0	++
Pons	Locus coeruleus	+	0	+++
	Pontine nucleus	0	0	+
Medulla oblongata	Dorsal nucleus of vagal nerve	0	0	+++
	Inferior olivary nucleus	+	0	++

Severity was graded as follows: 0, none; +, mild; ++, moderate; +++, severe

Globose-type neurofibrillary tangles (NFTs) were observed in the substantia nigra using hematoxylin and eosin (H&E) staining and IHC against phosphorylated tau (1:4000, mouse monoclonal, clone AT-8; Thermo Scientific, Rockford, IL, USA). H&E staining showed that the subthalamic nucleus and the internal and external segments of the globus pallidus were preserved (Fig. 3G). However, a moderate number of NFTs and coiled bodies were detected using IHC against phosphorylated tau and Gallyas–Braak staining (Fig. 3H). NFTs and coiled bodies were present to a similar degree in the globus pallidus and midbrain tegmentum but few were seen in the superior frontal gyrus (Fig. 3I–K). A few tufted astrocytes were observed in the putamen, midbrain and precentral gyrus (Fig. 3L). In the oculomotor nucleus and the Edinger–Westphal nucleus, a very small number of NFTs were observed. Although the overall PSP-related tau pathology was mild, it met the neuropathological diagnostic criteria for PSP (Table 1) [3]. Contusions were confirmed in the right inferior temporal gyrus. The age-related NFT pathology corresponds to Gallyas–Braak stage III [7]. IHC for Aβ (1:1000, mouse monoclonal, clone 12B2; IBL, Gunma, Japan) revealed no senile plaques. IHC for phosphorylated TDP-43 (1:4000, rabbit polyclonal; Cosmo Bio, Tokyo, Japan) showed no immunoreactivity. No argyrophilic grains were observed in Gallyas-Braak staining.

## Discussion and conclusions

Despite the absence of supportive imaging study and the potential influence of traumatic brain injury on the neurological examination, this case exhibited core clinical features of DLB, including fluctuating cognition, visual hallucinations, and parkinsonism, fulfilling the criteria for probable DLB [1]. Neuropathologically, the diagnosis of DLB was further supported by typical α-synuclein pathology involving the brainstem and limbic system [1]. Postural instability and repeated falls are supportive clinical features of DLB; however, repeated unprovoked falls within 3 years of disease onset are a core clinical feature of PSP and an important feature in the differential diagnosis for another Parkinson syndrome [2]. Although multiple factors may influence the risk of falls, including advanced age, impaired cardiac function, chronic subdural hematoma, and autonomic dysfunction because of Lewy body pathology in the sympathetic ganglia, repeated head trauma resulting in cerebral contusions raises concern that additional pathological processes may have contributed to the severe postural instability [4]. In this patient, tufted astrocytes, a pathological hallmark of PSP, were observed in the precentral gyrus, putamen, and midbrain. In addition, NFTs were distributed in the substantia nigra, subthalamic nucleus, and globus pallidus, thus fulfilling the minimal pathological criteria for the diagnosis of PSP [3]. As the patient died only 3 months after the onset of gait disturbance and frequent fall, the overall mild pathological burden may be interpreted as representing incipient PSP [8].

Several autopsy studies of patients with LBD and PSP have described cases with the coexistence of LBD and PSP tau pathology [9–13]. Tufted astrocytes, which are pathological hallmarks of PSP, were also found in 10 of 60 LBD autopsy cases [10]. Further, Lewy bodies have been found in 10–30% of patients with PSP [9, 11–13]. One study showed that the age at death of patients with LBD and tufted astrocytes was significantly higher than that of patients without tufted astrocytes [10]. Additionally, the average disease duration in patients with PSP with LBD was reported to be significantly shorter than that in patients with LBD alone and was similar to that in patients with PSP alone [9]. Despite these observations, definitive clinical features suggesting the coexistence of LBD and PSP have not yet been clearly identified. One report showed that the number of Lewy bodies was not higher in patients with PSP than in healthy controls [12]. In large cohorts, the coexistence of LBD and PSP may be an incidental finding.

Some case reports have detailed the clinical presentations of patients with the coexistence of PSP and DLB. Mori et al. [5] described an autopsy case of a patient with PSP and Lewy bodies who exhibited memory impairment and psychiatric symptoms, including silence and gloom, hallucinations, Parkinsonism, and supranuclear gaze palsy. The patient presented with clinical features consistent with DLB, such as hallucinations and apathy, along with the characteristics of PSP, including supranuclear gaze palsy. Kobylleckie et al. [6] described a case of coexisting LBD and PSP that was difficult to differentiate from Alzheimer’s disease and suggested that the combination of tau and Lewy body pathologies in the limbic regions contributed to an atypical amnestic phenotype. The patient presented with rapid eye movement sleep behavior disorder, cognitive fluctuations, and mild Parkinsonism, which are consistent with DLB, whereas the occurrence of falls was more consistent with PSP. The present case was an autopsy case with an approximately 1-year clinical course and exhibited core features of DLB, such as fluctuating cognition, visual hallucinations, and Parkinsonism. Further, the present patient had a marked tendency to fall, resulting in cerebral contusion and traumatic subarachnoid hemorrhage, which indicates severe postural instability, a characteristic of PSP. Neuropathological examinations revealed findings consistent with typical DLB, supporting the notion that the clinical symptoms of the patient during life were primarily attributable to DLB. Furthermore, we presume that the coexistence of PSP may have contributed to the early and severe postural instability experienced by the patient. This case illustrates that the coexistence of different neurodegenerative pathologies, including DLB and PSP, can result in diverse clinical manifestations.

Recent diagnostic criteria have divided PSP into eight clinical subtypes based on their clinical manifestations [2]. Postural instability is regarded as an important component of the diagnostic criteria for PSP and is a characteristic feature observed predominantly in the PSP–Richardson’s syndrome and PSP–postural instability subtypes. A correlation has been reported between the distribution of tau pathology and the PSP clinical subtype [14]. Although the overall degree of PSP-related tau pathology in the present case was mild, the frontal lobe showed minimal involvement, whereas the subcortical nuclei and midbrain tegmentum were more severely affected; this distribution is consistent with the PSP–postural instability or PSP–Richardson’s syndrome subtypes [14]. The mild tau pathology may reflect an early stage of PSP, as the patient was autopsied following an unexpected death [8]. Furthermore, tau pathology in the midbrain tegmentum in patients with PSP may be related to postural instability [14]. The mesencephalic locomotor region, composed of the pedunculopontine nucleus and cuneiform nucleus in the midbrain tegmentum, is a key region for locomotor control (Fig. 2C) [15]. Both tau and α-synuclein pathologies were observed in the cuneiform nucleus in the present case (Fig. 3F and J). Pathology in the pedunculopontine nucleus is more likely to be associated with postural instability and falls in Parkinson’s disease [16], although it was not identified in this case because of the absence of a pedunculopontine nucleus specimen. We believe that the coexistence of tau and α-synuclein pathologies in the mesencephalic locomotor region may play a key role in the early onset of severe gait disturbances, postural instability, and falls, which can lead to cerebral contusion or traumatic subarachnoid hemorrhage.

Antemortem diagnosis of concomitant pathologies remains challenging. Biomarker-based diagnostic approaches, including positron emission tomography, may provide potential utility; however, further autopsy-based studies are required to establish their reliability in the presence of concomitant pathologies.

## Data Availability

All data supporting the findings of the present study have been included in the manuscript.

## Abbreviations

DLB: Dementia with Lewy bodies
LBD: Lewy body disease
PSP: Progressive supranuclear palsy

## References

[1] McKeith IG, Boeve BF, Dickson DW, Halliday G, Taylor JP, Weintraub D, et al. Diagnosis and management of dementia with lewy bodies: fourth consensus report of the DLB consortium. Neurology. 2017;89:88–100. 10.1212/wnl.0000000000004058. 28592453 10.1212/WNL.0000000000004058 PMC5496518

[2] Höglinger GU, Respondek G, Stamelou M, Kurz C, Josephs KA, Lang AE, et al. Clinical diagnosis of progressive supranuclear palsy: the movement disorder society criteria. Mov Disord. 2017;32:853–64. 10.1002/mds.26987. 28467028 10.1002/mds.26987 PMC5516529

[3] Roemer SF, Grinberg LT, Crary JF, Seeley WW, McKee AC, Kovacs GG, et al. Rainwater charitable foundation criteria for the neuropathologic diagnosis of progressive supranuclear palsy. Acta Neuropathol. 2022;144:603–14. 10.1007/s00401-022-02479-4. 35947184 10.1007/s00401-022-02479-4 PMC9468104

[4] Tolosa E, Garrido A, Scholz SW, Poewe W. Challenges in the diagnosis of parkinson’s disease. Lancet Neurol. 2021;20:385–97. 10.1016/s1474-4422(21)00030-2. 33894193 10.1016/S1474-4422(21)00030-2 PMC8185633

[5] Mori H, Yoshimura M, Tomonaga M, Yamanouchi H. Progressive supranuclear palsy with lewy bodies. Acta Neuropathol. 1986;71(3–4):344–6. 10.1007/bf00688061. 3026136 10.1007/BF00688061

[6] Kobylecki C, Thompson JC, Robinson AC, Roncaroli F, Snowden JS, Mann DM. Concomitant progressive supranuclear palsy and lewy body pathology presenting with circumscribed visual memory loss: A clinicopathological case. Brain Pathol. 2024;34:e13219. 10.1111/bpa.13219. 37927160 10.1111/bpa.13219 PMC11189767

[7] Crary JF, Trojanowski JQ, Schneider JA, Abisambra JF, Abner EL, Alafuzoff I, et al. Primary age-related tauopathy (PART): a common pathology associated with human aging. Acta Neuropathol. 2014;128:755–66. 10.1007/s00401-014-1349-0. 25348064 10.1007/s00401-014-1349-0 PMC4257842

[8] Yoshida K, Hata Y, Kinoshita K, Takashima S, Tanaka K, Nishida N. Incipient progressive supranuclear palsy is more common than expected and may comprise clinicopathological subtypes: a forensic autopsy series. Acta Neuropathol. 2017;133 5:809–23. 10.1007/s00401-016-1665-7. 28064358 10.1007/s00401-016-1665-7

[9] Uchikado H, DelleDonne A, Ahmed Z, Dickson DW. Lewy bodies in progressive supranuclear palsy represent an independent disease process. J Neuropathol Exp Neurol. 2006;65:387–95. 10.1097/01.jnen.0000218449.17073.43. 16691119 10.1097/01.jnen.0000218449.17073.43

[10] Hishikawa N, Hashizume Y, Yoshida M, Niwa J, Tanaka F, Sobue G. Tuft-shaped astrocytes in lewy body disease. Acta Neuropathol. 2005;109:373–80. 10.1007/s00401-004-0967-3. 15668789 10.1007/s00401-004-0967-3

[11] Mori H, Oda M, Komori T, Arai N, Takanashi M, Mizutani T, et al. Lewy bodies in progressive supranuclear palsy. Acta Neuropathol. 2002;104:273–8. 10.1007/s00401-002-0555-3. 12172913 10.1007/s00401-002-0555-3

[12] Tsuboi Y, Ahlskog JE, Apaydin H, Parisi JE, Dickson DW. Lewy bodies are not increased in progressive supranuclear palsy compared with normal controls. Neurology. 2001;57:1675–8. 10.1212/wnl.57.9.1675. 11706110 10.1212/wnl.57.9.1675

[13] Gearing M, Olson DA, Watts RL, Mirra SS. Progressive supranuclear palsy: neuropathologic and clinical heterogeneity. Neurology. 1994;44:1015–24. 10.1212/wnl.44.6.1015. 8208392 10.1212/wnl.44.6.1015

[14] Koizumi R, Akagi A, Riku Y, Miyahara H, Sone J, Tanaka F, et al. Correlation between clinical and neuropathological subtypes of progressive supranuclear palsy. Parkinsonism Relat Disord. 2024;127:106076. 10.1016/j.parkreldis.2024.106076. 38494398 10.1016/j.parkreldis.2024.106076

[15] Noga BR, Whelan PJ. The mesencephalic locomotor region: beyond locomotor control. Front Neural Circuits. 2022;16:884785. 10.3389/fncir.2022.884785. 35615623 10.3389/fncir.2022.884785 PMC9124768

[16] Karachi C, Grabli D, Bernard FA, Tandé D, Wattiez N, Belaid H, et al. Cholinergic mesencephalic neurons are involved in gait and postural disorders in Parkinson disease. J Clin Invest. 2010;120:2745–54. 10.1172/jci42642. 20628197 10.1172/JCI42642 PMC2912198

